# Clinical and Phenotypic Characteristics of Early-Onset Inflammatory Bowel Disease: A Five-Year Observational Study

**DOI:** 10.3390/children12070952

**Published:** 2025-07-18

**Authors:** Ivan S. Samolygo, Marina A. Manina, Ekaterina A. Yablokova, Pavel A. Stribul, Alexander V. Novikov, Anton S. Antishin, Albina S. Pestova, Alexander S. Tertychnyy, Daniel Munblit, Svetlana I. Erdes

**Affiliations:** 1 Department of Propaedeutics of Children’s Diseases, N.F. Filatov Clinical Institute of Children’s Health, I.M. Sechenov First Moscow State Medical University (Sechenov University), 8/2 Trubetskaya Str., 119991 Moscow, Russia; manina12345@mail.ru (M.A.M.); pestova_a_s@staff.sechenov.ru (A.S.P.);; 2 Department of Childhood Diseases, N.F. Filatov Clinical Institute of Child Health, I.M. Sechenov First Moscow State Medical University (Sechenov University), 8/2 Trubetskaya Str., 119991 Moscow, Russia; 3 Institute of Clinical Morphology and Digital Pathology, I.M. Sechenov First Moscow State Medical University (Sechenov University), Lane, 1, Build. 1. Abrikosovskiy, 119435 Moscow, Russia; 4 Department of Internal, Occupational Diseases and Rheumatology, N.V. Sklifosovsky Institute of Clinical Medicine, I.M. Sechenov First Moscow State Medical University (Sechenov University), 8/2 Trubetskaya Str., 119991 Moscow, Russia; 5 Care for Long Term Conditions Division, Florence Nightingale Faculty of Nursing, Midwifery and Palliative Care, King’s College London, Strand, London WC2R 2LS, UK; 6 Department of Paediatrics and Paediatric Infectious Diseases, Institute of Child’s Health, I.M. Sechenov First Moscow State Medical University (Sechenov University), 8/2 Trubetskaya Str., 119991 Moscow, Russia

**Keywords:** EO-IBD, ulcerative colitis, Crohn’s disease, pediatric-onset IBD

## Abstract

**Background:** Inflammatory bowel diseases with an early-onset form (EO-IBDs) make up a special disease group with certain clinical and phenotypic characteristics. This article discusses the features of such early onset in a group of children, based on five years of monitoring a registry of children with IBD from a specialized center. **Methods:** This retrospective single-center cohort study included pediatric patients diagnosed with EO-IBD between 2019 and 2024. Clinical, laboratory, and endoscopic data were collected from medical records, including fecal calprotectin, inflammatory markers, disease activity indices, and endoscopic severity scores. Localization was classified according to the Paris system, and histological activity was assessed using the IBD-DCA score. **Results:** There were 20 patients with ulcerative colitis (UC) and 17 with Crohn’s disease (CD). Clinical activity was moderate or high (*p* = 0.179). UC was more characterized by diarrhea and rectal bleeding. CD was more often accompanied by abdominal pain, weight loss, and fever. In total, 82.4% of patients with CD had an inflammatory form. UC-like intestinal lesion was typical of both nosologies—L3 64.7% and E4 60% forms in CD and UC, respectively. Morphological activity was moderate for both nosologies (*p* = 0.54). IBD-U was present in 43.2% of patients. The median time after which it was possible to diagnose UC was 24 weeks (IQR 20–48) and 40 weeks (IQR 30–45.5) for CD (*p* = 0.56). **Conclusions:** Our study confirms the presence of characteristic signs of EO-IBD development, such as a frequent family history of IBD, high or moderate clinical activity during diagnosis verification, colon damage, and a high frequency of extraintestinal manifestations.

## 1. Introduction

Inflammatory bowel diseases (IBDs) comprise a group of immuno-mediated diseases, including nosologies such as ulcerative colitis (UC), Crohn’s disease (CD), and unclassified forms of IBD (IBD-U). Over the past few decades, the incidence of IBD in pediatric populations has trended upwards, particularly among patients under the age of 6 [1,2,3]. The classification of these patients as a separate group occurred in 2014 [4] due to clinical features and a better understanding of the course of the disease. Very-early-onset inflammatory bowel disease (EO-IBD) is characterized by pancolitis, resistance to standard therapies, and a high risk of adverse phenotypes and complications [4,5]. Incidence in this group is lower than in the general child population with IBD, at 0.4–4.37 per 100,000 children [6,7]. Such differences are attributed to difficulties in interpreting the clinical, endoscopic, and morphological patterns in children under 6 years old, leading to an increase in IBD-U and delay in diagnosis and pathogenetic therapy [5]. In addition, specific monogenic features determine the course of the disease [8]. P. Parente et al. suggest that 15 to 30% of early forms of IBD are monogenic and accompanied by mutations in genes associated with primary immunodeficiencies (PIs) [5]. PIs can “hide” behind the mask of monogenic IBD, not only delaying diagnosis for decades but also complicating specific treatment [9].

The purpose of our study was to analyze the clinical, laboratory, and endoscopic features of the early onset of IBD in children at a gastroenterological center in the Russian Federation.

Novelty: A high frequency of family history of IBD has been demonstrated. Differences in the appearance of EIMs in relation to the time of occurrence of intestinal manifestations are shown. The features of IBD onset in a group of young children have been demonstrated.

## 2. Materials and Methods

This retrospective single-center cohort study included patients who were examined from 2019 to 2024 in connection with the diagnosis of EO-IBD. A total of 5242 people were examined at our center during this time period, of which 218 (4.2%) were diagnosed with IBD and 37 (16.9%) with EO-IBD. In all patients, the onset of IBD occurred before the age of 6 years; intestinal infections were ruled out in all children. IBD was diagnosed according to the ESPGHAN Revised Porto criteria 2014 [10]. Demographic (age and sex, body weight and height), clinical, and laboratory data were taken from patients’ outpatient and inpatient records. Intestinal (abdominal pain, changes in stool amount and shape, pathological admixtures in feces, nutritional deficiencies, and perianal manifestations of CD) and extraintestinal manifestations were assessed. The levels of fecal calprotectin (for children over 4 years old), C-reactive protein, albumin, and hepatic transaminases were used as laboratory markers for activity. The clinical activities of CD and UC were assessed using the Pediatric Crohn’s Disease Activity Index (PCDAI) and Pediatric Ulcerative Colitis Activity Index (PUCAI), respectively [10]. Additionally, the presence of a family predisposition to autoimmune and auto-inflammatory diseases and the timing of their onset were taken into account. The Simple Endoscopic Score for Crohn’s Disease (SES-CD) and Ulcerative Colitis Endoscopic Index of Severity (UCEIS) were used to assess endoscopic activity and UC severity, respectively [11]. The localization of the lesion was assessed using the Paris Classification [10]. Adverse phenotypes were identified in patients with CD [11]. To assess morphological changes (histological activity) in biopsy samples of the colon mucosa, we used the inflammatory bowel disease—distribution, chronicity, activity score (IBD-DCA score) [12]. There were no symptoms suspicious of primary immunodeficiency in our patients, including the presence of recurrent otitis media, sinus infections, ineffective courses of antibacterial therapy and recurrent pneumonia. In all patients diagnosed before the age of 1.5 years, the levels of total IgA, IgM, and IgG, subpopulations of T and B cells, and T-cell receptor excision circle (TREC) repertoires were examined to exclude PI (primary immunodeficiency). By locus sequencing, we excluded X-linked lymphoproliferative syndrome type 2 (XLP2), capping protein regulator and myosin 1 linker 2 (CARM1L2) protein deficiency, mevalonate kinase deficiency, and IL-10 receptor deficiency. We also ruled out the activation of the phosphoinositide 3-kinase-δ (PI3Kδ) gene, signal transducer and activator of transcription 1 (STAT 1) gene defects, and superkiller viralicidic activity 2-like (SKIV2L) syndrome [13]. This study was approved by the Local Ethics Committee, with reference number 15–24, dated 6 June 2024. Informed consent from the study participants was sought prior to recruitment. Statistical analyses were carried out using the Microsoft Excel 2022 software package (macOS version 14) and jamovi 2.5.7. The normality of the distribution of quantitative variables was checked using the Shapiro–Wilk test. Quantitative data are presented in the form of means ± standard deviation (SD, with a normal distribution), or medians with interquartile ranges (IQRs) with distributions other than normal; qualitative data are presented as fractions (as percentages). For quantitative data, the statistical significance of between-group differences was assessed using the Mann–Whitney test, taking into account abnormal distributions for most indicators. For qualitative studies, the χ^2^–Pearson method was used, along with the calculation of the odds ratio (OR) and 95% confidence intervals (95% CI). Spearman’s correlation analysis was employed to determine the significance of the relationship between quantitative features that had an abnormal distribution. The construction of a predictive model of the probability of a certain outcome was performed using the logistic regression method. The Nigelkirk coefficient R^2^ served as a measure of certainty indicating the part of the variance that can be explained using logistic regression. Related nominal aggregates were compared using McNemar’s test. Test results were considered significant at *p* < 0.05 levels.

## 3. Results

### 3.1. Characteristics of Patients

This study included 37 patients, 17 with CD and 20 with UC, whose IBD onset occurred before the age of 6 years inclusive. The UC group consisted of 11 boys and 9 girls, while the CD group consisted of 11 boys and 6 girls. The median (Me) age of patients at diagnosis of IBD was 5 years for UC (IQR 3–6), and 5 years for CD (IQR 4–6). The groups were comparable in terms of sex (*p* = 0.55) and age (*p* = 0.81). The interval between the onset of the first intestinal manifestations of the disease before diagnosis did not significantly differ (*p* = 0.74): 10 months in patients with UC (IQR 6.25–18) and 8.5 months in patients with CD (IQR 5.5–15.8).

### 3.2. Heredity

Four patients with UC (20%) had a positive family history for IBD. Three parents (two mothers and one father) of children with UC had confirmed UC. One mother of a child with UC had confirmed CD. Three patients with CD (17.6%) had a parent with the same diagnosis. Most parents developed CD or UC in adulthood (>30 years), while two parents of children with CD and UC developed CD during adolescence (>10 years).

### 3.3. Body Weight and Height

Growth retardation (SDS < −2) was observed in four 6-year-old boys with CD. A decreasing body weight (BW) was typical for both patient groups. Among patients with UC, severe body weight deficiency (SDS < −3) was not observed; in patients with CD, weight gain retardation (SDS < −2) was more common (*p* = 0.019). In three patients, body weight deficit was noted (SDS −2–−1). In the CD group, four patients had severe BW deficit (SDS < −3), one had a BW deficit with SDS < −2, and three with SDS −2–−1.

### 3.4. Clinical and Laboratory Data

For patients with CD, the most characteristic symptoms were abdominal pain (64.7%) and weight loss (29.4%); fever was only present in this group (35.3%). UC was more often accompanied by diarrhea (90%), and blood in feces (85%). Episodes of defecation at night were noted only in patients with UC (45%). The onset of both diseases did not significantly differ: 17 (85%) patients with UC and 11 (64.7%) with CD had primary chronic onset. Clinical activity at the stage of disease onset was often moderate or high for both nosologies—PUCAI 37.5 (IQR 20–46.3) and PCDAI 25 (IQR 20–30) (Table 1).

The analysis of endoscopic video imaging protocols revealed a high incidence of UC-like intestinal lesions in both groups of patients—pancolitis (60%) and colitis (64.7%) in children with UC and CD, respectively. The most common endoscopic finding among patients with UC was erosion (75% vs. 41.2% in CD; *p* = 0.037). Ulceration was significantly more common in CD (70.6% vs. 30.0% in UC; *p* = 0.014). The endoscopic activity of ulcerative colitis was often moderate, 14 out of 20 (70%), and in 6 out of 17 cases (30%), it was low. In CD, low activity was detected in 5/17 (29.4%) cases; there were 6 (35.3%) cases each of moderate and high activity, respectively. Endoscopy of the upper gastrointestinal tract was performed in all patients. No endoscopic features suspicious for Crohn’s disease were identified, such as multiple deep transverse and longitudinal erosive or ulcerative lesions, strictures, or submucosal edema. Mild mucosal defects of the esophagus, consistent with reflux disease, were observed in 10 patients (27%). The phenotype of the CD course was dominated by non-stenosing and non-penetrating forms (B1)—14/17 (82.4%). Three cases had complicated phenotypes—two stenosing forms (B2) and one penetrating form (B3) with the formation of external fistula connections to paraproctium. The formation of an intra-abdominal infiltrate was not observed.

In addition to the main intestinal symptoms, we analyzed the presence of extraintestinal manifestations (EIMs) and determined how they related to the main clinical symptoms. EIMs were observed in 7/20 (35%) patients with UC and 7/17 (41.2%) with CD. The spectrum of EIMs is presented in Table 2. The combination of several EIMs in one patient was found only among patients with CD, of whom there were two. The most common EIM in both groups was peripheral arthritis—three (10%) and five (29.4%) in UC and CD, respectively (*p* = 0.29). At the same time, peripheral arthritis was significantly more often noted after the appearance of intestinal manifestations (*p* < 0.001). Uveitis was characteristic of patients with CD only; aphthous stomatitis was also more common in this group (23.5% vs. 5% in UC, *p* = 0.22). Regarding intestinal manifestations, the occurrence of aphthous stomatitis was not reliably obtained (*p* = 0.32). Primary sclerosing cholangitis was more typical of children with UC (10% vs. 5.9%, *p* = 0.65). As the first sign of the disease, EIMs were noted in two people from each group. In all other cases, EIMs appeared accompanied by intestinal symptoms.

**Figure 1:**
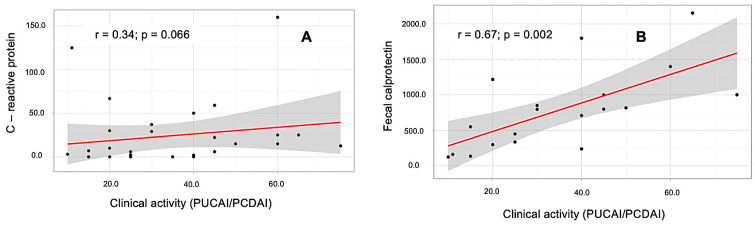
The results of the correlation analysis of the relationship of clinical activity (PUCAI/PCDAI) and CRP (**A**), and FCP (**B**).

To determine the laboratory inflammatory activity of the disease, we evaluated the values of C-reactive protein (CRP). An increase in CRP levels of more than 5 mg/L was observed in 13/20 of children with UC (Me 11.35 (IQR 0.1–26.23) mg/L) and in 8/17 of children with CD (Me 5 (IQR 1.4–29) mg/L); no significant differences were found (*p* = 0.8). We did not find a relationship between the level of clinical activity of the disease (PUCAI/PCDAI) and the level of CRP (*p* = 0.066; r = 0.34) (Figure 1A). An immunological study was conducted on the level of FCP in children over 4 years of age [14]. FCP was determined in 12 patients with UC and 14 patients with CD (there were no significant differences between the groups (*p* = 0.11)). An increase in FCP levels >200 mcg/g [15] was detected in 10 patients with UC, and in 12 patients with CD. We established a relationship between FCP level and clinical activity (PUCAI/PCDAI) (*p* = 0.002; r = 0.67); see Figure 1B. Hypoalbuminemia (<35 g/L) was observed in two children with CD, who had a severe body weight deficit. An increase in hepatic transaminase levels of >1 normal was observed in four patients with UC and one with CD. PSC-related liver damage was found in all of these patients (*p* < 0.001).

In some cases, interpreting the endoscopic findings was difficult due to pancolitis. In these cases, patients were diagnosed with IBD-U. In the UC group, nine (45%) patients had a history of IBD-U; among children with CD, seven (41.2%) had a history with IBD-U. When observing these patients, we determined the time (in weeks) after which it was possible to make a final diagnosis. The median time after which UC was determined was 24 weeks (IQR 20–48), and for CD, it was 40 weeks (IQR 30–45.5) (*p* = 0.56). When determining the relationship between the history of IBD-U and further development of adverse phenotypes (B2, B3) among CD patients, we did not obtain convincing results (*p* = 0.15).

### 3.5. Morphological Characteristics

**Figure 2:**
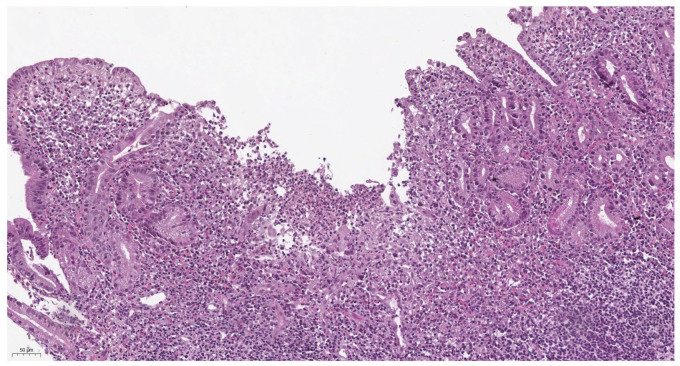
(50 μm H&E) Noteworthy budding, localized distortion of crypts within lamina propria, primarily in the basal regions, chronic pronounced inflammatory infiltration, occasional intraepithelial leukocytes are visible. Please note the artificial desquamation of the surface epithelium, which should not be mistaken for genuine erosion. The alterations correspond to a severity of 4.1 on the Heboes scale, as assessed by the IBD-DCA score: D2 C2 A1.

**Figure 3:**
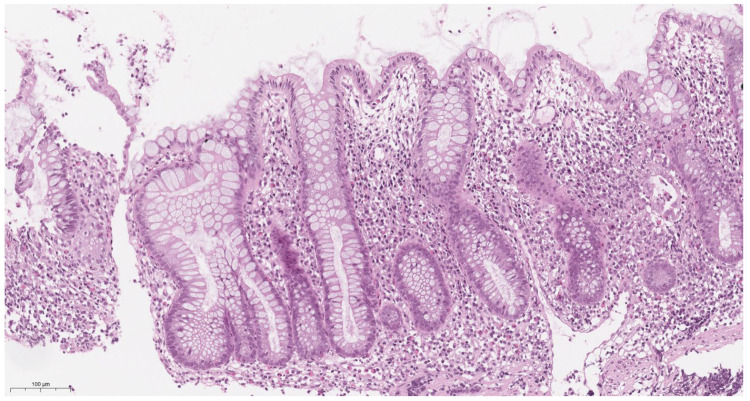
(100 μm H&E) Shortening, focal distortion, and separation of most crypts in the basal sections (D2 and C1 according to the IBD-DCA score) is visible.

According to the results of our study, there were no significant differences between histological activity in Crohn’s disease and ulcerative colitis (*p* = 0.54). For both nosologies, histological activity was moderate and amounted to 3.06 ± 1.64 and 2.75 ± 1.41, respectively (*p* = 0.54). However, we noted that morphological changes in ulcerative colitis are more characterized by crypt distortion, moderate lymphoplasmacytic inflammatory infiltration (C1), and the presence of intraepithelial neutrophils (A1) (Figure 2). In contrast, basal lymphoplasmasocytosis (C2) and crypt abscesses and erosions (A2) are more often detected in Crohn’s disease, and single-epithelial-cell granulomas were detected in several cases (Figure 3).

### 3.6. Therapy

All UC children (100%) and three (17%) CD children received 5-Aminosalicylic acid (5-ASA) therapy. In total, 28 (75.7%) children with EO-IBD (12 (60%) with UC (E4 localization), 16 children (94%) with various forms of CD (L1–L3, p, B1–B3) required induction steroid treatment—oral glucocorticosteroids (1 mg/kg according to a standard induction regimen [16]. The rate of clinical remission achieved with glucocorticosteroid induction therapy was 60.7% (17/28 children). However, we noted a high proportion of steroid dependence, 50% (14/28 children with EO-IBD). A total of 19 children (51.3%)—10 with UC and 9 with CD (*p* = 0.402)—received azathioprine as immunosuppressive therapy.

Anti-TNF therapy (infliximab or adalimumab) was required in 22 patients (59.5%) with EO-IBD, including 12 children with UC and 10 with CD (*p* = 0.94). The median duration of biologic therapy was 15 months (IQR 11.5–21.5). It was used as first-line therapy in three children with CD presenting with “B2”/“B3” phenotypes. In 19 (86.4%) patients, biologic therapy was initiated due to an insufficient response to conventional treatment and the development of steroid dependency. In 22.7% of cases (5 out of 22 children), the escalation of biologic therapy was required (Me 26.5 months (IQR 21–28)) due to secondary loss of response to anti-TNF treatment. During the observation period, no switches to second-line biologic agents were reported among children with EO-IBD. One patient with CD and perianal disease required surgical intervention with seton placement.

**Figure 4:**
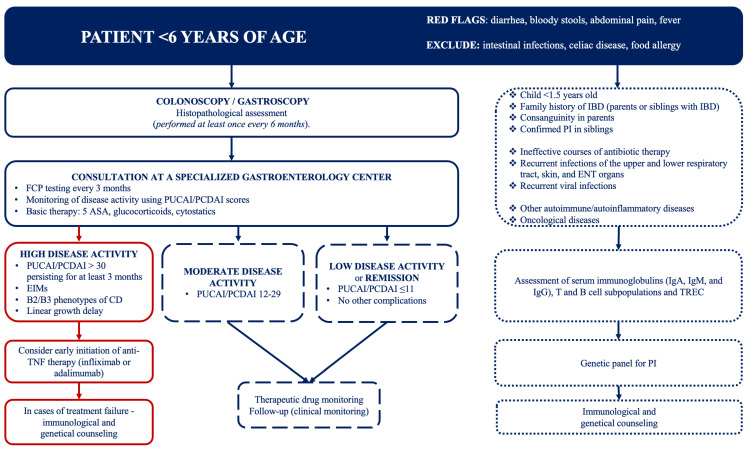
Diagnostic and therapeutic algorithm for the management of children with EO-IBD. Abbreviations: IBD—inflammatory bowel diseases; PI—primary immunodeficiency; ENT—ear, nose, and throat; EIMs—extraintestinal manifestations; FCP—fecal calprotectin; 5-ASA—5-aminosalicylic acid; CD—Crohn’s disease; TREC—T-cell receptor excision circles; TNF—tumor necrosis factor; PUCAI—Pediatric Ulcerative Colitis Activity Index; PCDAI—Pediatric Crohn’s Disease Activity Index.

To optimize medical care for children with EO-IBD, we suggest an algorithm summarizing our center’s approach to the diagnosis and management of EO-IBD (Figure 4).

## 4. Discussion

This single-center retrospective study included 37 children with EO-IBD at the onset stage. The number of patients with UC and CD did not significantly differ. UC was characterized using loose stools and rectal bleeding, while CD was more often accompanied by abdominal pain, weight loss, and fever. Rectal bleeding and diarrhea were among the most common intestinal manifestations for both nosologies, which is confirmed by data from other studies and is explained by the phenotypic feature of EO-IBD—pancolitis in UC and CD [6,17,18]. At the same time, abdominal pain occurred in slightly more than half of the patients in the CD group. Studies show that this is more common in older children (>10 years old) [6,18,19]. This may be due to difficulties in expressing pain in young children and difficulties in formulating complaints. The appearance of extraintestinal manifestations of IBD was noted in most cases after the onset of intestinal symptoms. Weight loss, as well as single or multiple extraintestinal manifestations, is characteristic of children whose CD debuted at the age > 10 years [16]. In our study, extraintestinal manifestations were noted in 45% of participants (*p* = 0.72). This reflects their high prevalence but has little effect on establishing the main diagnosis because they appear in most cases after the intestinal manifestations. Peripheral arthritis was the most common extraintestinal manifestation in both groups. The clinical activity of disease at onset was moderate to high in our cohort of patients. This is consistent with data from Aloi et al. [17].

Growth retardation and severe body weight deficiency were noted only among patients with CD. We attribute this to delayed diagnosis due to the possible absence of specific complaints, as well as the onset of EIMs.

Inflammatory activity was assessed using CRP levels, as well as fecal analysis for FCP. An increase in CRP levels was observed in half of the children in both groups. Some data show that changes in the concentration of CRP better reflect the state of the intestine in patients with CD than with UC [20]. We performed fecal analysis for FCP in patients older than 4 years old due to limitations in the result interpretation for younger children [18].

In our study, as in the studies by Bequet E. et al. [6] and Aloi M. et al. [17], a high incidence of UC-related intestinal lesions was noted among patients with CD: 64.7% had the L2 form according to the Paris classification [10], L1 and L3 lesions were rarer and accounted for less than 40% of all cases, and L4 lesions were not detected. Our data are consistent with the observations of other authors in that the lesions of the terminal parts of the ileum and the upper gastrointestinal tract are mainly characteristic of children over 10 years of age [17]. In UC, the characteristic type of lesion was E3, which also demonstrates the total nature of the colon lesion in this age group. We would like to emphasize the nature of colon lesions in EO-IBD as one of the key features of the phenotype at this age. The presence of a UC-like character in intestinal lesions in UC and CD may indirectly indicate the absence of mutations in NOD2/CARD15 genes, which was demonstrated in a study by Arie Levine et al. [21]. Adverse CD phenotypes (B2 and B3, and their combination) were identified in 17.6% of patients, which is a rate as low as in similar studies [6].

Moderate histopathological activity was observed in both UC and CD. UC was characterized by a superficial lesion, with slight distortion of the crypts, as well as intraepithelial infiltration by neutrophils. The histological pattern of CD was characterized by deep lesion, with basal lymphoplasmocytosis; the presence of >2 neutrophils in one field of view, in lamina propria; and signs of crypt abscesses and erosions. Epithelioid granulomas have been found in several cases. Conversely, our work evaluated changes in the onset of disease, which, for this reason, may be relatively nonspecific. At the same time, we believe that the assessment of morphological changes in each case should be carried out in conjunction with clinical and laboratory data analysis. In some cases, doing so helps not so much in the differential diagnosis of Crohn’s disease and ulcerative colitis but in differentiating the detected changes from those in drug and infectious lesions of the colon. An integrated interdisciplinary approach also helps with the early diagnosis of PI that occurs under the “mask” of IBD [5,22]. The accuracy of the diagnosis, in our opinion, is undoubtedly increased by performing a ladder biopsy of the colon and a mandatory biopsy of the terminal ileum.

One of the main difficulties in diagnosis is still the presence of IBD-U. In our study, as well as in the studies by Mamula et al. and Aloi et al. [17,23] the interpretation of the results from 43% of patients was difficult at the onset of the disease. We attribute this to the characteristics of the phenotype of the colon and the difficulties in interpreting endoscopic examinations. It is noteworthy that the time frame after which it was possible to determine the diagnosis from IBD-U to UC or CD was different: it took longer to diagnose CD.

One of the limitations of our study was the small sample size of patients who underwent genetic sequencing to exclude PI. Only 2 out of 37 patients (5.4%) underwent genetic testing and were excluded for XLP2, CARM1L2 protein deficiency, mevalonate kinase deficiency, IL-10 receptor deficiency, activated PI3Kδ syndrome, STAT1 gene defects, and SKIV2L syndrome. Currently, approximately 70 genes responsible for immunodeficiencies presenting under the “mask” of EO-IBD have been described in the literature [24]. Diagnosing these syndromes remains challenging not only due to atypical clinical presentations but also because of the high cost associated with diagnostic methods. In the future, attention should be paid both to the identification of new PI-related genes and to more accessible “markers” that could suggest EO-IBD onset. One such marker may be IL-17A. Recent studies by Rudbaek et al. in neonatal patients have shown that IL-17A may be associated with the early onset of EO-IBD [25]. However, further research is needed to evaluate this and other interleukin candidates as potential prognostic biomarkers.

It is important to identify children with potential monogenic IBD and PI among those with EO-IBD, as treatment strategies may differ from conventional IBD management approaches [13]. Some studies have demonstrated a poor response of EO-IBD to standard therapies, including 5-ASA, glucocorticosteroids, and immunosuppressants, both during induction and maintenance phases [17,26,27]. In our study, a high need for corticosteroid therapy (75.7%) was observed in the EO-IBD group, along with an exceptionally high rate of steroid resistance (50%) within this cohort. The use of immunosuppressive agents from various classes (azathioprine, methotrexate, tacrolimus, cyclosporine A) in EO-IBD requires strict indications, as these drugs can cause bone marrow and T-lymphocyte suppression [28]. In this study, azathioprine therapy was required in every second child with EO-IBD. The insufficient efficacy of conventional therapy and the more severe disease course necessitated the use of anti-TNF biologic agents (infliximab or adalimumab) in 59.5% of patients. Previous studies have demonstrated the high efficacy of anti-TNF therapy in inducing and maintaining remission in children with EO-IBD [29,30]. However, numerous reports highlight altered pharmacokinetics of anti-TNF agents in younger children, often requiring therapy escalation or switching to second-line biologics [27,30]. In our study, therapy escalation was necessary in 22.7% of patients. These findings underscore the need for personalized treatment approaches in EO-IBD, including the use of therapeutic drug monitoring (TDM) to allow timely dose adjustments.

## 5. Conclusions

Our study touches on the clinical and diagnostic aspects of IBD with early onset in children and confirms the presence of its characteristic features in young children, such as the phenotypic features of the onset of CD and UC, the presence of high or moderate clinical activity at diagnosis verification, pancolits, widespread extraintestinal manifestations, etc. However, for children with EO-IBD, we also faced many unresolved problems: difficulties in verifying the diagnosis at the early stages of the nosological forms, which results in the diagnosis of IBD-U; the aggressive onset and course of the disease requiring other therapeutic algorithms, with the exclusion of PI, etc.

## Figures and Tables

**Table 1:** General characteristics of patients.

Category	n	UC	CD	OR	95% Cl	*p*-Value
Number of patients with IBD	37	20	17	n/a	n/a	n/a
Gender						
Male n (%)	22	11 (55)	11 (64.7)	n/a	n/a	0.55
Family history for IBD	7	4 (20)	3 (17.6)	n/a	n/a	0.86
Age at the time of diagnosis, Me (IQR), years	37	5 (3–6)	5 (4–6)	n/a	n/a	0.81
The age of the first symptoms (mean + SD), months	37	43.9 ± 19.1	45.9 ± 17.7	n/a	n/a	0.74
Mass-growth characteristics						
Height, SDS < −2	4	n/a	4 (23.5)	n/a	n/a	n/a
Body weight						
SDS < −3	4	n/a	4	n/a	n/a	0.02 *
SDS < −2	7	6 (30)	1 (5.9)	n/a	n/a	
SDS < −2–−1	6	3 (15)	3 (17.6)	n/a	n/a	
BMI (mean ± SD), kg/m^2^	37	13.8 ± 1.38	13.6 ± 1.32	n/a	n/a	0.62
Clinical characteristics, n (%)						
Abdominal pain	23	12 (60)	11 (64.7)	0.82	0.22–3.12	0.77
Diarrhea	30	18 (90)	12 (70.6)	3.75	0.62–22.6	0.13
Rectal bleeding	28	17 (85)	11 (64.7)	0.32	0.067–1.57	0.15
Defecation at night	9	9 (45)	n/a	n/a	n/a	n/a
Fever	6	0	6 (35.3)	n/a	n/a	n/a
Anemia	1	1 (5)	0	n/a	n/a	n/a
Extraintestinal manifestations	7	7 (35)	7 (41.2)	n/a	n/a	1
Primary chronic onset	28	16 (80)	12 (70.6)	n/a	n/a	0.44
Subacute onset	9	4 (20)	5 (29.4)	n/a	n/a	
*Laboratory*						
Fecal calprotectin (mean ± SD), mcg/g	22	721.49 ± 365.33	1070.25 ± 559.09	n/a	n/a	0.11
CRP Me (IQR)	37	11.35 (0.1–26.23)	5 (1.4–29)	n/a	n/a	0.8
Albumin (mean ± SD), g/L	37	42.5 ± 4.18	43.5 ± 6.64	n/a	n/a	0.22
Endoscopic characteristics, n (%)						
CD, localization n (%)	17	n/a	n/a	n/a	n/a	n/a
L1	1	n/a	1 (5.9)	n/a	n/a	n/a
L2	11	n/a	11 (64.7)	n/a	n/a	n/a
L3	5	n/a	5 (29.4)	n/a	n/a	n/a
CD, phenotypes n (%)						
E1	14	n/a	14 (82.4)	n/a	n/a	n/a
E2	2	n/a	2 (11.8)	n/a	n/a	n/a
E3	1	n/a	1 (5.8)	n/a	n/a	n/a
UC, localization n (%)						
E2	4	4 (20)	n/a	n/a	n/a	n/a
E3	4	4 (20)	n/a	n/a	n/a	n/a
E4	12	12 (60)	n/a	n/a	n/a	n/a
Endoscopic findings						
Ulcers	18	6 (30)	12 (70.6)	5.60	1.36–23.1	0.02 *
Aphthae	12	5 (25)	7 (41.2)	2.10	0.52–8.51	0.29
Erosion	22	15 (75)	7 (41.2)	4.29	1.06–17.4	0.04 *
Limited continuous inflammation of the intestinal mucosa	8	6 (30)	2 (11.8)	0.31	0.054–1.81	0.18
Microabscess	5	4 (23.5)	1 (5)	0.25	0.025–2.49	0.21
PCDAI (Me (IQR)	17	n/a	25 (20–30)	n/a	n/a	n/a
PUCAI (Me (IQR)	20	37.5 (20–46.3)	n/a	n/a	n/a	n/a
Duration of diagnosis IBD-U, Me (IQR), week	16	24 (20–48)	40 (30–45.5)	n/a	n/a	0.56

Notes to Table 1. (*p* < 0.05 is considered statistically significant. Significant results are marked with an asterisk (*); n/a—no data available; UC—ulcerative colitis; CD—Crohn’s disease; IBD-U—undifferentiated colitis; BMI—body mass index; CRP—C-reactive protein; g/L—grams per liter; mcg/g—micrograms per gram; PCDAI—Pediatric Crohn’s Disease Activity Index; PUCAI—Pediatric Ulcerative Colitis Activity Index; mean ± SD—mean ± standard deviation; OR—odds ratio; Me (IQR)—median (interquartile range)).

**Table 2:** The spectrum of EIMs.

	UC			CD			*p*-Value
	EIMs, n	Before the Appearance of Intestinal Manifestations, n	After the Appearance of Intestinal Manifestations, n	EIMs, n	Before the Appearance of Intestinal Manifestations, n	After the Appearance of Intestinal Manifestations, n	
P/a	2	1	2	5	1	4	<0.001 *
Erythema nodosum	2	0	2	1	0	1	n/a
Uveit	0	0	0	1	0	1	n/a
A/s	1	1	0	4	1	3	0.32
PSC	2	0	2	1	0	1	n/a

Notes to Table 2. (*p* < 0.05 is considered statistically significant. Significant results are marked with an asterisk (*); n/a—no data available; EIMs—extraintestinal manifestations; UC—ulcerative colitis; CD—Crohn’s disease; P/a—peripheral arthritis; A/s—aphthous stomatitis; PSC—primary sclerosing cholangitis).

## Data Availability

The data presented in this study are available on request from the corresponding author. The data are not publicly available due to patient privacy concerns and institutional data protection policies.
